# Description and disposition of home patients with colorectal cancer accessing a practical, complications related medication therapy management service

**DOI:** 10.3389/fonc.2025.1595010

**Published:** 2025-07-17

**Authors:** Qinbo Wang, Yuan Zhou, Hua Li, Yingjuan Ou, Jiaxi Fei, Xia Wu, Junrong Chen, Xiaoyan Li

**Affiliations:** ^1^ Department of Pharmacy, The Sixth Affiliated Hospital, Sun Yat-Sen University, Guangzhou, China; ^2^ Department of Graceland Medical Center, The Sixth Affiliated Hospital, Sun Yat-Sen University, Guangzhou, China; ^3^ Biomedical Innovation Center, The Sixth Affiliated Hospital, Sun Yat-sen University, Guangzhou, China; ^4^ Department of Gastroenterology, The Sixth Affiliated Hospital, Sun Yat-Sen University, Guangzhou, China; ^5^ Department of General Practice, The Sixth Affiliated Hospital of Sun Yat-Sen University, Guangzhou, China

**Keywords:** colorectal cancer, MTM, complication, adverse effect, KPA

## Abstract

**Objective:**

To explore a practical complications related Medication Therapy Management (MTM) service for colorectal cancer patient which based on take home cancer drugs (THCDs), and minimize the occurrence of unexpected events by reducing complications and adverse reactions in home therapy.

**Method:**

A total of 144 patients with colorectal cancer (CRC) who underwent home cancer drugs treatment for the first time met the include criteria from July 1, 2023 to July 31, 2024. They were divided into control group and MTM intervention group randomly, MTM intervention group conducted with three courses of MTM intervention, and control group adapt with three times of conventional follow up. We compared patient characteristics, complications, adverse effects, and knowledge-practice-attitude (KPA) results.

**Results:**

Among them, 119 patients were enrolled. There were significant differences regard of cancer pain, insomnia, anxiety, and defecation disorder (p<0.05); Multivariate analysis results showed that pain, chemotherapy-induced nausea or vomiting (CINV), and defecation disorder were independent factors for unscheduled hospital admission (p<0.05); There were significant differences regard of adverse effects for home medication patient which include jaundice, hypo leukocytosis, limb edema, and fatigue (p<0.05); MTM intervention group showed better feedback than control group in Attitudes and practice Toward screening (p<0.05).

**Conclusion:**

MTM demonstrates significant clinical benefits in colorectal cancer (CRC) patients by effectively reducing the incidence of treatment-related complications, including nausea and vomiting (CINV), abdominal pain, and insomnia. Furthermore, it contributes to decreased rates of unplanned hospitalization and enhances key patient outcomes (KPA), warranting further investigation and clinical application in CRC management.

## Introduction

Colorectal cancer (CRC) stands as one of the most prevalent malignant tumors of the digestive system, ranking third in global incidence and second in mortality rates. According to the latest statistics from the International Agency for Research on Cancer (IARC) under the World Health Organization, the global burden of CRC reached 1,933,600 new cases in 2020 (1), with China alone reporting 517,100 new cases in 2022 (2). Over the past decades, significant advancements in treatment modalities have led to a steady improvement in survival rates (3, 4), particularly through the development of novel therapeutic agents and optimized chemotherapy regimens. The current standard of care for CRC involves a multidisciplinary approach, combining surgical intervention with adjuvant therapies including radiotherapy, chemotherapy, targeted therapy, and integrated traditional Chinese medicine. Clinical evidence demonstrates that patients undergoing chemotherapy achieve significantly higher 3- and 5-year overall survival (OS) and progression-free survival (PFS) rates compared to non-chemotherapy groups (5, 6)). However, the cytotoxic nature of chemotherapeutic agents not only targets malignant cells but also adversely affects healthy tissues, leading to a spectrum of treatment-related complications (7, 8). This is particularly concerning for home-based care, as studies indicate that over 50% of terminal cancer patients require hospital readmission due to acute complications arising during home treatment (9, 10).

The paradigm of cancer management has evolved with medical advancements, establishing take-home cancer drugs (THCDs) as an integral component of CRC treatment protocols. Modern pharmacotherapy for CRC has become increasingly sophisticated, encompassing not only antineoplastic agents but also comprehensive supportive care medications. While existing research in China has predominantly focused on clinical characteristics, healthcare utilization patterns, and intravenous chemotherapy regimens (11), there remains a notable gap in understanding the management of gastrointestinal complications during home-based treatment. Specifically, insufficient attention has been given to critical issues such as cancer-related pain, nutritional deficiencies, CINV, defecation disorder, and sleep disturbances. These unaddressed complications frequently necessitate hospital readmissions for symptom management, significantly compromising patients’ quality of life and treatment adherence.

Emerging evidence highlights the efficacy of medication therapy management (MTM) in optimizing patient outcomes (12). This approach, particularly when implemented through home-based pharmaceutical care programs, has demonstrated significant benefits in medication safety evaluation and adverse drug reaction management (13, 14). Notably, systematic management of THCDs has been associated with reduced 90-day readmission rates (15). Despite these advancements, there remains a global knowledge gap regarding the implementation of MTM services for CRC patients and the optimization of THCDs programs. Therefore, this study aims to investigate home-based medication strategies to effectively manage treatment-related complications, mitigate adverse drug reactions, enhance treatment compliance, and prevent unexpected clinical events through comprehensive drug therapy management services within the THCDs framework.

## Methods

### Study design and patients

A total of 144 patients who came to the Sixth Affiliated Hospital of Sun Yat-sen University for the treatment of colorectal cancer from January 2023 to Aug 2023 were selected. The trial protocol was approved by the Ethics Committee of the Sixth Affiliated Hospital of Sun Yat-sen University, approval number 2022ZSLYEC-616. all patients provided written informed consent prior to initiation of any study treatment. The inclusion criteria were: (1) Patients aged 18 or above were diagnosed by pathological examination as colorectal cancer patients in TNM stages II to IV (American Cancer Federation 8^th^ Edition); (2) Colorectal cancer patients first time received THCDs therapy; (3) Complete clinical data and postoperative follow-up data. Exclusion criteria: (1) Patients with other malignant tumors; (2) End-stage patients dead or give up drug treatment midway; (3) Patients with communication difficulties and difficulty in obtaining relevant data. They were divided into control group and MTM intervention group according to random number table method, with 72 cases in each group. The control group was included in general discharge guidance, and the observation group was included in MTM service of take-home cancer drugs.

### MTM procedure

#### Medication therapy management

**Figure 1:**
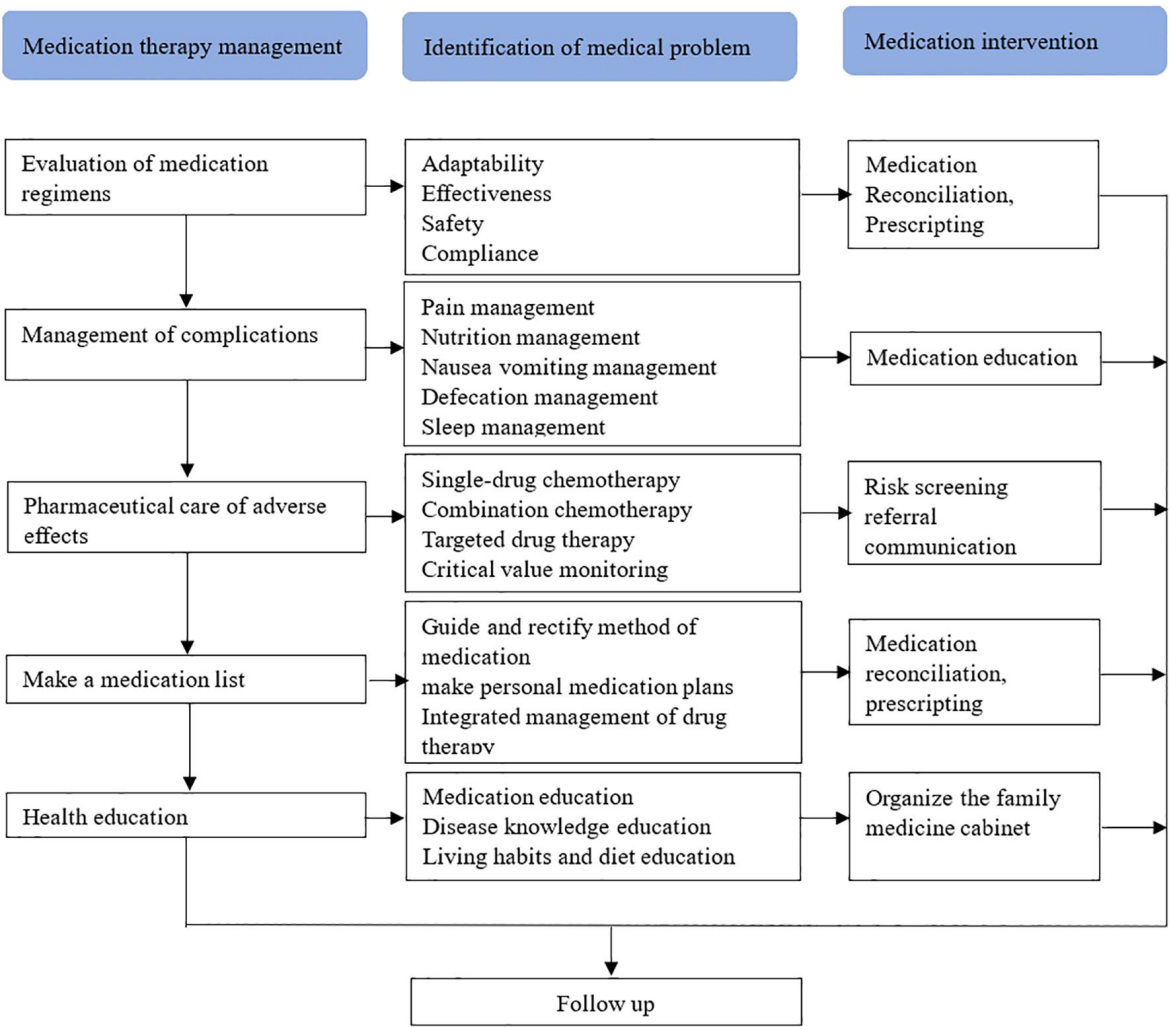
Intervention strategies include the evaluation of medication regimen, management of complications, pharmaceutical care of adverse effects, make a medication list, and health education, aims to identify the medical problems of take-home cancer drugs, thereby improving medication compliance and preventing medication errors.

Medication therapy management (MTM) intervention strategies were developed by integrating core MTM elements with disease characteristics (**Figure 1**). Three rounds of MTM intervention were conducted after the first, third, and sixth course of chemotherapy, each including three sections.

#### Evaluation of medication regimens

Section 1 involves the comprehensive documentation of patients’ current drug use, including chemotherapy drugs, targeted drugs, and immune drugs. The Screening Tool of Older Person’s Prescriptions (STOPP) and Screening Tool to Alert doctors to Right Treatment (START) are utilized to evaluate the indications, effectiveness, safety, and compliance of these medications based on evidence-based practice and individual patient circumstances.

#### Management of complications

In Section 2, the focus is on evaluating potential issues related to chemotherapy for colorectal cancer and associated complications such as cancer pain, nutrition, nausea/vomiting, bowel movements, and insomnia. This includes providing health education, medication guidance, and psychological counseling for patients in order to manage complications effectively.

#### Pharmaceutical care of adverse effects

Section 3 details the recording of adjusted medication regimens for patients with colorectal cancer along with management plans for disease complications and concurrent conditions. It also involves categorizing the patient’s personal drug plan list according to drug purpose while providing suggestions for proper medication use. For patients with improper drug utilization, recommendations are made for reorganization of their medication regimen through communication with specialists or attending doctor.

Following three rounds of Medication Therapy Management (MTM) intervention for colorectal cancer patients comes follow-up management.

### Conventional follow-up

Conventional follow up included the explanation of the treatment duration, hospitalization appointment, and therapy attention, also education of living habits and dietary precautions, Health & Nutrition suggestion, and reminded the patient of the return time. Three times of conventional follow up was adapt after each course of chemotherapy at first visit, third visit, and sixth visit.

### Statistical analysis

All statistical analyses were performed using SPSS statistical software system (Version 25.0, SPSS, Chicago). Frequency analysis was conducted to identify the general characteristics of the study patients, and a descriptive statistical analysis was conducted to identify the level of research variables. A paired sample t-test was conducted to verify the difference in the perception of the importance of MTM in oral drugs. The chi-square test was used to evaluate the percentage and difference between the two groups, and the Kruskal–Wallis test was used to compare the categorical data. Values of p<0.05 were considered statistically significant.

## Results

### Clinical demographics

A total of 119 patients with colorectal cancer were eligible among 144 participants met the inclusion criteria who underwent home cancer drugs treatment from January 1, 2023 to August 31, 2023, with 60 participants allocated to MTM intervention group (34 men, 26 women) and 59 participants allocated to Control group (39 men, 20 women), and the mean ages of the patients from MTM intervention group and Control group were 57.28 ± 12.81 and 59.03 ± 11.07 years respectively. There were no significant differences between the two groups in terms of age, BMI, cancer type, or therapeutic schedule (p>0.05; **Table 1**).

**Table 1:** Characteristics of the participating colorectal cancer patient.

Characteristic	MTM intervention group (n=60)	Controls (n=59)	*P* value
Age (years ± SD)	57.28 ± 12.81	59.03 ± 11.07	0.286
Gender			0.198
Male	34 (56.67%)	39 (66.10%)	
Female	26 (43.33%)	20 (33.90%)	
BMI (kg/m^2^)	21.52 ± 2.14	20.34 ± 1.82	0.215
Cancer type, n (%)			0.080
Colon cancer	17 (28.33%)	12 (20.34%)	
Sigmoid colon cancer	14 (23.33%)	18 (30.51%)	
Rectal cancer	29 (48.33%)	28 (47.46%)	
Rectosigmoid junction cancer	0 (0.00%)	1 (1.69%)	
Therapeutic schedule			0.055
Chemotherapy	23 (38.33%)	20 (38.90%)	
Chemotherapy with targeted therapy	5 (8.33%)	13 (22.03%)	
Chemotherapy with immunotherapy	29 (48.33%)	24 (40.68%)	
Other combination therapy	3 (5.00%)	2 (3.39%)	

MTM, medication therapy management. Data are presented as No. (%) or mean ± standard deviation.

### Complication symptoms for home medication patient with colorectal cancer

Patients with colorectal cancer experience a range of complications primarily affecting the gastrointestinal tract throughout disease progression and the post-surgical period. Significant differences were observed in terms of cancer pain, insomnia, malnutrition, CINV, anxiety, and defecation disorder (p<0.05; **Table 2**). In the MTM intervention group, a lower percentage of patients experienced pain (51.67% vs 76.27%), insomnia (41.67% vs 49.15%), malnutrition (58.33% vs 89.83%), CINV (30% vs 37.29%), anxiety (40% vs 44.07%), and defecation disorder (41.67% vs 67.80%) compared to the control group. There was significantly difference between the two groups in terms of unscheduled hospital admission (UHA), which 21.67% of the patients in the MTM intervention group and 30.51% in the control group (p<0.05).

**Table 2:** Evaluation of complications between two groups.

Parameter	MTM intervention group (n=60)	Controls (n=59)	t	p
Pain	31 (51.67%)	45 (76.27%)	-6.047	0.026
Mild	10 (16.67%)	12 (20.34%)		
Moderate	12 (20.00%)	17 (28.81%)		
Severe	9 (15.00%)	16 (27.12%)		
Insomnia	25 (41.67%)	29 (49.15%)	7.488	0.000
Malnutrition	35 (58.33%)	53 (89.83%)	-6.379	0.024
Mild	15 (25.00%)	25 (42.37%)		
Moderate	17 (28.33%)	19 (32.20%)		
Severe	3 (2.00%)	9 (15.25%)		
Nausea and vomiting	18 (30.00%)	22 (37.29%)	-2.045	0.045
Anxiety	24 (40.00%)	26 (44.07%)	6.761	0.000
Defecation disorder	25 (41.67%)	40 (67.80%)	-4.332	0.049
Constipation	18 (30.00%)	25 (42.37%)		
Ileus	1 (1.67%)	6 (10.17%)		
Diarrhea	6 (10.00%)	9 (15.25%)		
Others	4 (6.67%)	5 (8.47%)	-1.000	0.374
Total UHA (numbers)	5 ± 2.67	7 ± 3.51	-2.317	0.024

MTM, medication therapy management; UHA, unscheduled hospital admission. Data are presented as No. (%) or mean ± standard deviation.

### Multivariate analysis of complications during home medication treatment

Relationship between unscheduled hospital admission and complications were conducted by multivariate analysis. Pain, insomnia, malnutrition, CINV, anxiety and defecation disorder were selected as significant risk factors of UHA (**Table 3**). Multivariate analysis results showed that pain, CINV, and defecation disorder were independent factors for unscheduled hospital admission (p<0.05).

**Table 3:** Multivariate analysis of the risk of unscheduled hospital admission (UHA).

Parameter	n	R-Squared	Adjusted R-Squared	Odds ratio (95%-CI)	P value
Pain	86	0.106	0.098	0.659(0.560-0.758)	0.000
Insomnia	54	0.118	0.110	0.651(0.550-0.752)	0.000
Malnutrition	104	0.024	0.015	0.575(0.465-0.686)	0.095
Nausea and vomiting	66	0.021	0.013	0.567(0.465-0.679)	0.115
Anxiety	50	0.024	0.015	0.569(0.463-0.674)	0.094
Defecation disorder	73	0.038	0.030	0.588(0.485-0.691)	0.033
Others	9	0.029	0.021	0.359(0.209-0.509)	0.161

MTM, medication therapy management; UHA, unscheduled hospital admission. Data are presented as No. (%) or mean ± standard deviation.

### The adverse effects between two groups

**Figure 2:**
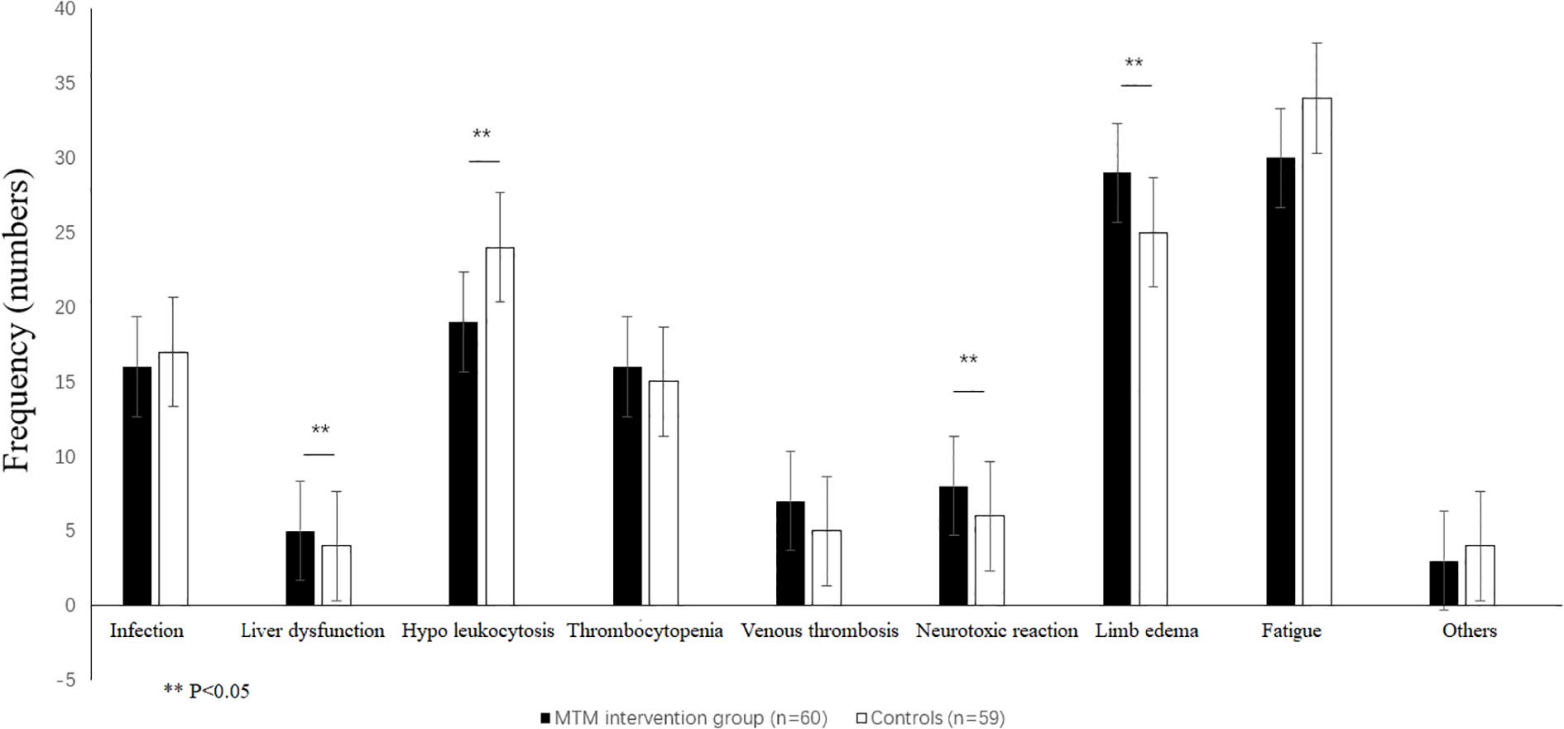
Patient reported adverse reactions included treatment infections, jaundice, hypo leukocytosis, thrombocytopenia, venous thrombosis, peripheral neuritis, limb edema, and purpose unclear. **p<0.05, by a t-test.

Colorectal cancer patients commonly receive various drug combinations that frequently lead to adverse drug reactions such as infection(urinary tract infection, pneumonia, surgery site infection), decreased liver function(jaundice), bone marrow suppression (primarily decreased white blood cells, platelets), venous thrombosis, limb edema, fatigue, and gastrointestinal reactions (inability to eat properly along with symptoms like nausea or vomiting), neurotoxic reactions (sensory disorders in extremities or abnormal sensations accompanied by painful cramps or cold sensitivity), skin manifestations oral mucositis impairment, the adverse effects. The most common adverse effects were fatigue, limb edema, hypo leukocytosis, and occurred 57.63%, 48.33%, and 40.68% in control group, and significantly decreased in MTM intervention group which were 50.00%, 42.37%, and 31.67%. There were significantly difference between the two groups in terms of liver dysfunction, hypo leukocytosis, limb edema, and fatigue (p<0.05; **Table 4**; **Figure 2**).

**Table 4:** Adverse effects of home medication patient with colorectal cancer.

Parameter	MTM intervention group (n=60)	Controls (n=59)	t	p
Infection	16 (26.67%)	17 (28.81%)	0.608	0.605
Urinary tract infection	6 (10.00%)	2 (3.39%)		
Pneumonia	9 (15.00%)	15 (25.42%)		
Surgery site infection	1 (1.67%)	0 (00.00%)		
Liver dysfunction	5 (8.33%)	4 (6.78%)	1.000	0.037
Hypo leukocytosis	19 (31.67%)	24 (40.68%)	-2.317	0.024
Thrombocytopenia	16 (26.67%)	15 (25.42%)	1.000	0.321
Venous thrombosis	7 (11.67%)	5 (8.47%)	1.549	0.172
Neurotoxic reaction	8 (13.33%)	6 (10.17%)	1.528	0.170
Limb edema	25 (42.37%)	29 (48.33%)	2.054	0.045
Fatigue	30 (50.00%)	34 (57.63%)	-2.054	0.045
Others	3 (5.00%)	4 (6.78%)	-1.000	0.321

MTM, medication therapy management. Data are presented as No. (%) or mean ± standard deviation.

### Questionnaire results of KPA from participants

A total of 119 questionnaires were sent out and returned, we counted “Yes” responses n (%). The Knowledge and views regarding the colorectal cancer and therapy rate in Group A was higher than group B (p<0.001). MTM intervention group showed better feedback than control group in Attitudes and practice Toward screening (p<0.05; **Table 5**).

**Table 5:** Patient’ attitudes and practice toward colorectal cancer screening and self-examination.

Question	MTM group (n=60)	Controls (n=59)	t	P value
Knowledge and views regarding the clinical presentation			6.120	0.000
Do you consider yourself an informed person, with awareness of the colorectal cancer?	28 (46.67%)	20 (33.90%)		
According to your knowledge, is there a proven link between the presence of adenocarcinoma polyps in the large intestine and the possibility of colorectal cancer development?	26 (43.33%)	12 (20.34%)		
Do you think that colorectal cancer can be an inherited disease?	22 (36.67%)	14 (23.73%)		
Do you think inflammatory bowel diseases can be linked to the development of colorectal cancer?	34 (56.67%)	22 (37.29%)		
Do you think that the type of diet can affect the development of colorectal cancer?	40 (66.67%)	28 (47.46%)		
Do you know what a screening is?	29 (48.33%)	16 (27.12%)		
Does colorectal cancer in your immediate family put you at an increased risk for developing colorectal cancer?	42 (70.00%)	35 (59.32%)		
Is fecal occult blood test helpful in detecting colorectal cancer?	38 (63.33%)	21 (35.60%)		
Do you think that taking regular small doses of aspirin may protect against colorectal cancer?	32 (53.33%)	16 (27.12%)		
Do you think colorectal cancer is a malignant neoplastic disease?	57 (95.00%)	57 (96.61%)		
Do you know what an intestinal stoma (fecal fistula) is?	32 (53.33%)	16 (27.12%)		
Do you pay attention to the appearance of stool, bearing in mind that a change in the appearance of stool or finding e g. blood in it may be one of colorectal cancer symptoms?	28 (46.67%)	28 (47.46%)		
Knowledge and views regarding the therapy			4.679	0.009
Can colorectal cancer be treated surgically?	53 (88.33%)	48 (81.36%)		
Do you know the drugs commonly used to treat colorectal cancer patients?	52 (86.67%)	25 (42.37%)		
Do you know how to use chemotherapy or targeted drugs and the course of treatment?	39 (65.00%)	18 (30.51%)		
Do you know the common adverse reactions and precautions of chemotherapy or targeted patient drugs?	31 (51.67%)	16 (27.12%)		
Do you know how to cope with complications of chemotherapy or targeted patients?	34 (56.67%)	17(28.81%)		
Attitudes and practice toward colorectal cancer screening			4.856	0.017
Have you ever looked for information about colorectal cancer?	37 (61.67%)	18 (30.51%)		
If necessary, would you agree to undergo colostomy to have permanent intestinal stoma if that would be required?	16 (26.67%)	7 (11.86%)		
Will you get regular colonoscopy screening?	50 (83.33%)	34 (57.63%)		
Do you agree with the statement that colorectal cancer can be completely cured in any case?	18 (30.00%)	10 (16.95%)		

MTM, medication therapy management. Data are presented as No. (%) or mean ± standard deviation.

## Discussion

The implementation of Take-Home Cancer Drugs (THCDs) has become indispensable in the management of various malignancies (16). However, patients undergoing home-based treatment frequently encounter challenges due to insufficient medical support and limited understanding of their therapeutic regimens, potentially leading to medication errors or inappropriate drug use. Medication Therapy Management (MTM) interventions encompass a comprehensive approach, including the management of comorbidities, monitoring of additional medications, surveillance for adverse events, assessment of potential drug interactions, patient education, guidance provision, adherence evaluation, and management of treatment-related toxicities (17, 18). Pain represents a predominant symptom in the CRC population, with epidemiological studies indicating a prevalence exceeding 70% (19–21). Chemotherapy-induced peripheral neuropathy frequently manifests through characteristic symptoms such as seizures and neuropathic pain (22). The constipating effects of opioid analgesics are well-documented, with literature reporting constipation incidence rates ranging from 40% to 90% among chemotherapy patients (23, 24). These findings are consistent with our observational data, which revealed constipation rates of 42.37% in the control group versus 30% in the MTM intervention group. The pathophysiology is further complicated by reduced oral intake secondary to chemotherapy-induced anorexia, nausea, and vomiting.

The MTM intervention demonstrated significant clinical efficacy in symptom management. Pain severity was markedly reduced, with 51.67% of intervention group patients reporting improvement compared to 76.27% in controls. Sleep disturbances and bowel dysfunction showed notable improvement, with 30% fewer patients experiencing insomnia (versus 37.29% in controls) and 41.67% fewer patients reporting defecation disorders (compared to 67.8% in controls). The intervention group also demonstrated reduced consultation rates for surgical complications including anastomotic leakage, hemorrhage, wound infections, and pulmonary infections. Statistical analysis revealed significantly lower incidence rates of pain, sleep disturbances, weight loss, nausea/vomiting, and anxiety in the MTM group (p<0.05), accompanied by reduced unscheduled hospital admissions. Multivariate regression analysis identified significant correlations between unscheduled admissions and the presence of pain, insomnia, or defecation disorders (p<0.05). The intricate interplay between pain-induced insomnia and gastrointestinal symptoms creates a complex clinical syndrome that substantially impairs quality of life (25, 26). This cyclical relationship suggests that therapeutic strategies should prioritize the simultaneous management of these interconnected symptoms while minimizing treatment-related adverse effects (19).

Common challenges in home medication management include dosing errors, non-adherence (manifesting as under- or over-medication), severe toxicities, and postoperative complications (27, 28). Adverse drug reactions can be objectively assessed through laboratory parameters and clinical manifestations. For instance, leukopenia predisposes patients to infections, thrombocytopenia may cause hemorrhagic complications, hepatic dysfunction can lead to jaundice, and hypoalbuminemia may present as peripheral edema, while immunological disturbances can manifest as cutaneous pruritus. Alarmingly, medication non-adherence contributes to approximately 125,000 annual deaths (29), with cancer patients demonstrating particularly low adherence rates to oral therapies (46%) (30). However, targeted interventions have shown efficacy in improving medication adherence among high-risk populations (31, 32). Our findings indicate that structured medication management significantly reduced the incidence of hepatic dysfunction, leukopenia, peripheral edema, and fatigue in home-treated patients (p<0.05).

Patients with colorectal cancer received personalized education on disease management and medication protocols, tailored to their educational background and comprehension abilities. The educational program encompassed a comprehensive range of topics, including risk factors, clinical manifestations, treatment options, drug indications and dosages, optimal timing of medication administration, necessary precautions, management of adverse drug reactions, handling of overdoses or missed doses, lifestyle recommendations, and monitoring frequency for symptoms and signs during medication use (33). To assess the impact of the intervention, surveys were conducted using key performance indicators (KPA), which included 12 statements related to clinical presentation and risk factors, 5 statements evaluating participants’ confidence and behavior, and 5 statements assessing attitudes toward colorectal cancer screening. The results revealed statistically significant differences between the MTM intervention group and the control group in terms of knowledge and understanding of clinical presentation and therapy, as well as attitudes toward colorectal cancer screening practices. These findings suggest that MTM intervention enhances patients’ disease knowledge, boosts confidence in rehabilitation, and improves treatment cooperation, ultimately leading to better prognosis (p<0.05).

MTM is designed to provide individualized pharmaceutical care throughout the entire course of home drug therapy. It aims to promote health literacy and improve medication compliance by offering evidence-based recommendations combined with practical application (34). The services include a holistic evaluation of all medications used by the patient, rather than focusing solely on drugs for a single condition. Additionally, MTM provides health education, manages complications, monitors for adverse reactions in colorectal cancer patients, and addresses concurrent symptoms during treatment. These issues are prioritized because they are more urgent and directly impact patients’ quality of life (35). The results demonstrate that this pharmaceutical care model not only reduces the incidence of adverse reactions but also minimizes complication rates, while emphasizing patient-centered care through personalized services.

## Conclusion

This study demonstrates that the application of MTM significantly reduces the incidence of THCDs-related complications such as CINV, abdominal pain, and insomnia, while also decreasing the rate of unscheduled hospital admissions. Furthermore, MTM enhances the KPA of colorectal cancer patients. However, the study has some limitations. First, the data were collected from a single center, this observation limits their applicability in a more heterogeneous international context, and future research should involve multi-center studies with larger populations to validate these findings. Additionally, due to the limited study duration, the relationship between complications and survival rates was not explored. Nevertheless, this study enrolled a substantial number of cases and included detailed follow-up parameters, enabling a comprehensive analysis. Therefore, the results and insights derived from this study remain valuable for advancing patient care in colorectal cancer management.

## Data Availability

The raw data supporting the conclusions of this article will be made available by the authors, without undue reservation.
